# Compositional, morphological, and physicochemical properties of starches from red adzuki bean, chickpea, faba bean, and baiyue bean grown in China

**DOI:** 10.1002/fsn3.865

**Published:** 2019-07-18

**Authors:** Zuosheng Zhang, Xiaolin Tian, Peng Wang, Hao Jiang, Wenhao Li

**Affiliations:** ^1^ Department of Arts and Sciences Yangling Vocational and Technical College Yangling China; ^2^ College of Food Science and Engineering Northwest A&F University Yangling China

**Keywords:** crystallinity, legume, pasting, swelling, thermal

## Abstract

Starches of four legume varieties grown in China were evaluated for composition, granule structure, turbidity, swelling power, solubility, and thermal and pasting properties. The similar granule shapes, surface fissures, polarized crosses, inner structure characteristics, and granule sizes could be observed among all legume varieties through a variety of microscopy techniques such as light microscopy, scanning electron microscopy, and confocal laser scanning microscopy. Amylose contents were in the range of 30.61%–33.55%. All of the starch varieties showed C‐type X‐ray pattern, but exhibited different relative crystallinity percentage. Significant differences were observed among starch varieties in swelling power, solubility, and light transmittance. Thermal analysis and pasting profile of legume starches showed that all the varieties’ differences are probably due to variation in amylose content. The thermal and pasting parameters of starches were evaluated using differential scanning calorimeter and Rapid Visco‐Analyser, respectively, and significant differences were observed in individual pasting and thermal parameters. The present study can be used for identifying differences between legume varieties for starch structural and physicochemical characteristics and could provide guidance to possible industries for their end use.

## 1 INTRODUCTION

Legumes such as the varieties and species of *Vigna angularis* L., *Cicer arietinum* L., *Vicia faba* L., and *Phaseolus vulgaris* L are the edible seeds of herbaceous plant belonging to the Fabaceae, Leguminosae, or Papilionaceae family. As important plant resources, there are about 16,000–19,000 species and approximately 750 genera in leguminosae family (Hoover, Hughes, Chung, & Liu, 2010). Beans play a major role in the human diet, because they are rich in proteins, carbohydrates, fiber, essential amino acids, minerals, and vitamin (especially the B‐complex vitamins). Besides, there are many biological active phytochemical components such as phenolic compounds (e.g., flavonoids, phenolic acids, anthocyanins, flavonol glycosides, tannins, and phytosterol), and lipophilic components such as tocopherols and carotenoids in beans, which have recently been related to the prevention of cancer, cardiovascular disease, obesity, and diabetes mellitus (Chen et al., 2015; Monk et al., 2015).

Starch is one of the most abundant substances in the legumes, accounts for roughly 45%–65% of the dry basis weight. As starch plays an important role in determining the quality of processed food, it is meaningful to study texture appearance, water‐holding capacity, thermal stability, and enzyme digestibility of processed foodstuff (Hoover et al., 2010; Kaur et al., 2013). According to the existing reports, there are many different properties between legume starches and cereal starches, such as high gelation temperature, high resistant starch and amylose content, resistance to shear thinning, more viscosity and lower swelling and breakdown during heating, fast retrogradation, and high elasticity of gel (Maaran, Hoover, Donner, & Liu, 2014; Ratnayake, Hoover, & Warkentin, 2002; Singh, Nakaura, Inouchi, & Nishinari, 2007).

There are about 20%–30% amylose (AP) and 70%–80% amylopectin (AM) in normal starch granule: Amylose is a linear chain of an hydroglucose units linked by a‐d‐1,4‐glucosidic linkage, while amylopectin is a branched polysaccharide containing thousands of short a‐d‐1,4‐glucan chains linked to each other by a‐d‐1,6‐linkages at branch points. These two kinds of polymers can be combined into five different starch structural levels including whole granule architecture (1–100 nm), growth rings (120–400 nm), blocklets (20–500 nm), amorphous and crystalline lamellae (9 nm), and AP and AM chains (0.1–1.0 nm; Pérez & Bertoft, 2010). Starch granules from different botanical origin can exhibit different kinds of shapes such as spheres, platelets, polygon, ellipsoids, and irregular tubules, and the diameters ranged from around 0.1–200 μm (Pérez & Bertoft, 2010). The native starch's internal architecture was made up of concentric alternating amorphous and semi‐crystalline growth rings (Singh et al., 2007). The lamellae crystalline regions are formed by double helices of AP side chains. There are three polymorphic forms, namely A‐type, B‐type, and C‐type. Most of the cereal starch has the A‐type polymorphs, and B‐type polymorphs can be easily found in tuber and high AM cereal starches. The C‐type polymorph (a mixture of A‐type and B‐type polymorphs) is considered to be intermediate between A‐type and B‐type polymorphs, which are generally found in most legume starches (Hoover et al., 2010).

**Figure 1:**
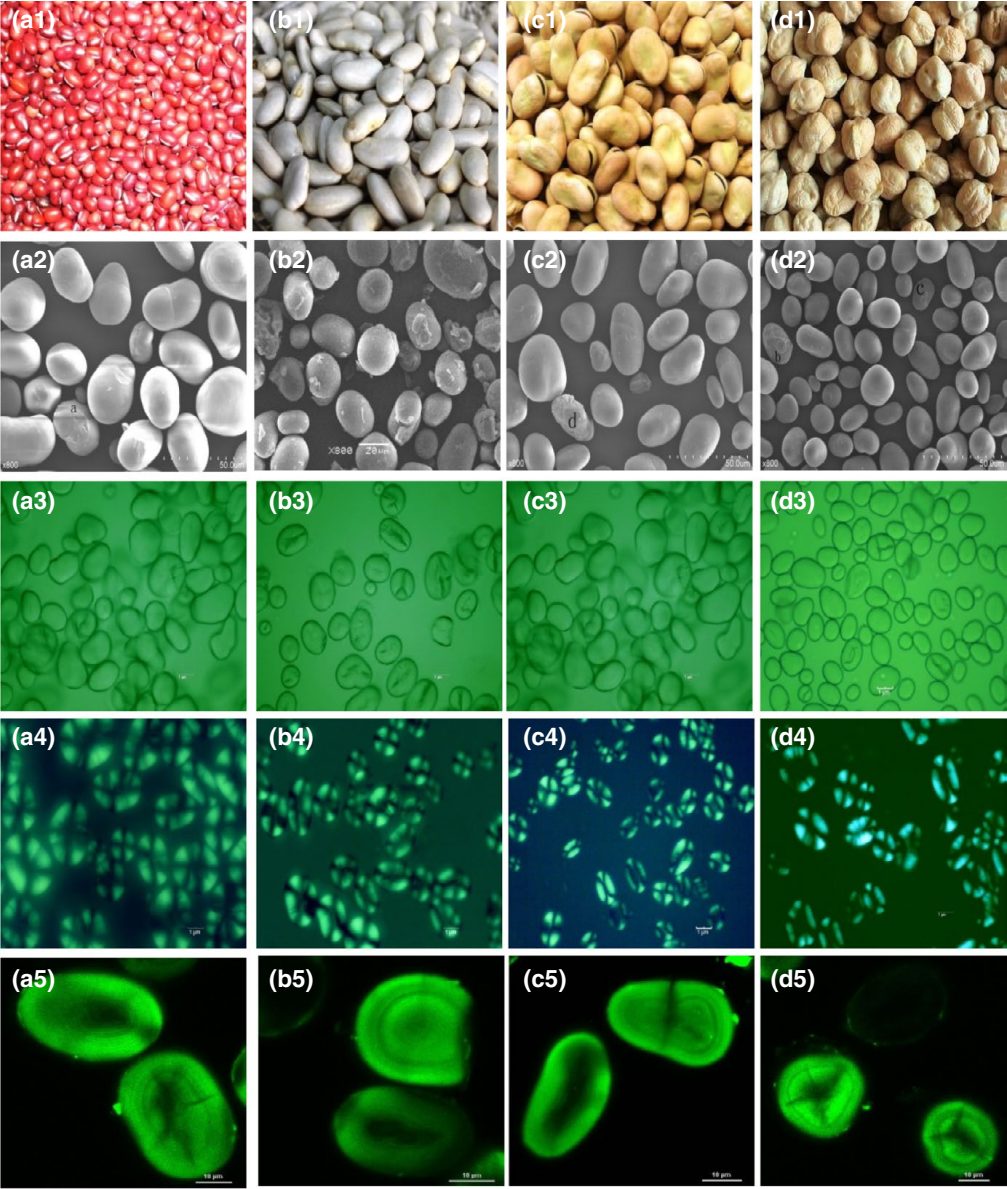
Legume seed photographs (1), scanning electron micrographs (SEM; 2), light microscope micrographs (LM; 3), polarized light micrographs (PLM; 4), and confocal laser scanning micrographs (CLSM; 5) of red adzuki bean (a), baiyue bean (b), faba bean (c), and chickpea (d) and their isolated starches

Physicochemical and structural properties of starches determine their applications in food and nonfood industries. In this study, four kinds of legumes including red adzuki bean (*V. angularis* L.; Figure 1a_1_), baiyue bean (*P. vulgaris* L.; Figure 1b_1_), Faba bean (*V. faba* L.; Figure 1c_1_), and chickpea (*C. arietinum* L.; Figure 1d_1_), which could commonly be found in China, were collected from their major producing region. Starches were isolated from these four kinds of beans, and their structural and physicochemical properties were investigated and compared. The results are expected to provide useful information for the applications of these starches in food and nonfood industries.

## 2 MATERIAL AND METHODS

### 2.1 Materials

The commercially mature red adzuki, faba bean, and chickpea seeds used in the study were purchased from a seed supplier at Mulei County in Xinjiang Province, China, while the baiyue bean seeds were obtained from Yushan County in Jiangxi Province, China. 8‐amino‐1, 3, 6‐pyrenetrisulfonic acid (APTS) and sodium cyanoborohydride were purchased from Sigma Chemical (St. Louis, MO, USA). All other chemicals and reagents used in experiments were of analytical grade.

### 2.2 Starch isolation

To isolate starches from four kinds of legumes, we adopt the method described by Singh, Kaur, Sandhu, and Guraya (2004), with proper modification. Briefly, firstly, seeds of legumes were washed with distilled water for several times until dust and other impurity substances were removed, and then, seeds were soaked in a solution which contains 0.16% sodium hydrogen sulfite for 24 hr at room temperature. After that, the steeped solution was drained off, and grains were ground in a laboratory blender. The obtained slurry passed through nylon cloth (100 mesh) successively, and the residue shall be washed by distilled water until no more starch was left. The material retained on the cloth all the time was discarded, and the filtrate slurry was allowed to stand for 1 hr. Afterward, the supernatant was removed by suction and the settled starch layer in distilled water was resuspended and the suspension was centrifuged at 2,200 *g* for 15 min, and then, the upper non‐white layer was scraped off. Starch was then collected and dried in an oven at 40°C for 12 hr. Then, the starches were smashed by universal mill grindings (100 mesh) and kept in a container at room temperature for further study.

### 2.3 Chemical composition analysis of legume starch

The starch, protein, lipid, and ash content were performed in terms of the standard AACC methods 76–13, 46–30, 30–25, and 08–01, respectively (AACC, 2000). The amylose content was determined by using the commercially available AM/AP assay kit (catalog: JKY/K‐AMYL, Megazyme International Ireland Ltd.). The results were reported on a dry basis.

### 2.4 Microscopy analysis

#### 2.4.1 Light microscopy observation

A 1:1 glycerol solution (glycerol/H_2_O, V/V) was prepared, and starch granules were suspended in it. Then, an aliquot of starch slurry was transferred onto a glass slide, and a coverslip was placed on the top of the sample for microscopic examination. A polarizing light microscope (DMBA400; Motic China Group Co., Ltd, Guangzhou, China) with a × 40 objective was used to observe birefringence of the starch granules. The microscope was also used without the polarizing filter to observe the appearance of the sample.

#### 2.4.2 Scanning electron microscopy (SEM) observation

Granular surface of legume starches was viewed with a scanning electron microscope (JSM–6360LV; JEOL, Japan) at an accelerating voltage of 20 kV. Starch samples which were dispersed evenly with a rubber suction bulb were mounted on an SEM stub with double‐sided adhesive tape and coated with gold in an argon atmosphere.

#### 2.4.3 Confocal laser scanning microscopy (CLSM) observation

The microstructure of starch granules was viewed by confocal laser scanning microscopy. The observation of each starch sample was conducted according to the reported procedure. Starch granules (10 mg) were suspended in 15 μl of freshly made APTS, and 15 μl of 1 M sodium cyanoborohydride solution was added. The reaction mixture was incubated at 30°C for 15–18 hr, and then, the granules were thoroughly washed five times with 1 ml of distilled water. The stained starch granules were then suspended in 20 μl of glycerol/water mixture (1:1, v/v) and then visualized using a Digital Eclipse C1plus CLSM system equipped with He/Ne and Ar lasers (Nikon Co., Ltd., Tokyo, Japan), and a stand for fixed fluorescent cell samples was used for the detection of the fluorescence signal from dye‐stained starch granules (Chen et al., 2011; Li, Xiao, Zhang, et al., 2014).

### 2.5 X‐ray diffraction analysis

X‐ray powder diffraction (XRD) measurements were performed using an X‐ray diffractometer (Rigaku D/max–2551/PC; Rigaku Corporation, Tokyo, Japan) with the following operating conditions: radiation source, CuKa; angle of diffraction scanned from 5 to 60°; step size, 0.02; and step time, 2 s. In order to avoid the negative effects of relative humidity on relative crystallinity, the starch samples were equilibrated in a 100% relative humidity chamber for 48 hr at room temperature in the first place.

### 2.6 Determination of swelling power and solubility

Swelling power (SP) and solubility (S) were measured following the method of Leach, McCowen, and Schoch (1959) at 50, 60, 70, 80, and 90°C for 30 min, respectively.

### 2.7 Determination of light transmittance (Turbidity)

The light transmittance of starches from different legume varieties was performed according to previous method (Craig, Maningat, Seib, & Hoseney, 1989).

### 2.8 Determination of thermal properties

Gelatinization characteristics of starch thermal properties were measured by a differential scanning calorimeter (DSC, Q2000; TA Co., New Castle, USA). Starch (3 mg) was put into the aluminum DSC pan and distilled water (12 μl) was added with a microsyringe and then sealed hermetically, reweighed, and allowed it to stand overnight at room temperature in order to attain an even distribution of water before DSC analysis. Subsequently, the samples were heated from 30 to 120°C at 10°C/min. Meanwhile, an empty pan was used as reference for all measurements. During the scans, the space surrounding the sample chamber was flushed with dry nitrogen to avoid condensation.

### 2.9 Determination of pasting properties

Pasting properties of starches were evaluated using a Rapid Visco‐Analyser (RVA) model Master (Newport Scientific, Pty Ltd, Australia). Firstly, starch (2.0 g, db) and deionized water (25.0 g) were put in the RVA canister, and the slurry was then manually homogenized using the plastic paddle to avoid lump formation before the RVA run. The starch slurry was heated from 50 to 95°C at the rate of 12°C/min, maintained at 95°C for 2.5 min, and then cooled to 50°C at the same rate. The speed of the paddle was 960 rpm for the first 10 s and then 160 rpm for the remainder of the experiment. Pasting temperature, peak viscosity (PV), breakdown viscosity, setback viscosity, and final viscosity were calculated.

### 2.10 Statistical analysis

All the experiments were performed at least in triplicate and experimental data also were analyzed using analysis of variance (ANOVA) and expressed as mean value ± *SD*. Duncan's least significant test was used to compare means at the 5% significance level. All statistical computations and analyses were conducted using SPSS 16.0 to compare Simple Pearson's correlation and regression for Windows.

## 3 RESULTS AND DISCUSSION

### 3.1 Proximate composition of legume starches

The chemical composition of legume starches is presented in Table 1. Significant differences could be observed in the proximate constituents of different legume varieties. The starch contents indicate the purity of the isolated starches, were reasonably high (>96%), and ranged from 96.58% to 98.79%. Because of the presence of insoluble protein, fine fiber, and minerals within the starch granules, it is difficult to obtain pure starch from legumes, but it could be decreased after sedimentation and co‐settled with the starch granules (Wang, Warkentin, Vandenberg, & Bing, 2014). The starch purity is generally influenced by the isolation method. The protein, fat, and ash contents were in the range of 0.23%–0.35%, 0.25%–0.42%, and 0.07%–0.14%, respectively, wherein baiyue bean showed significantly higher protein, fat, and ash contents value than rest of the legume values. The values of ash, fat, and protein content were in good agreement with the previous report on triangular pea, white pea, spotted colored pea and small white kidney bean, field pea, and lentil (Li, Xiao, Guo, et al., 2014; Wang et al., 2014). The low ash, fat, and protein contents in the isolated starches further indicated the high purity of the starches extracted from each sample.

**Table 1:** Chemical composition of various legume starches (w/w, %)^a^^,^ b

Varieties	Starch (%)	Protein (%)	Fat (%)	Ash (%)	Amylose (%)
Red adzuki	98.79 ± 0.08 a	0.23 ± 0.00 b	0.32 ± 0.03 b	0.08 ± 0.00 b	30.61 ± 0.37 b
Baiyue	96.62 ± 0.19 b	0.35 ± 0.01 a	0.42 ± 0.01 a	0.14 ± 0.00 a	32.64 ± 0.25 a
Faba bean	96.64 ± 0.06 b	0.30 ± 0.02 a	0.38 ± 0.02 a	0.07 ± 0.01 b	33.55 ± 0.98 a
Chickpea	96.58 ± 0.28 b	0.27 ± 0.02 b	0.25 ± 0.06 c	0.07 ± 0.01 b	32.61 ± 0.67 a

^a^Values reported as Mean ±* SD* of three replications and expressed in dry basis. ^b^Means in a column with the same letters are not significantly different at *p *<* *0.05.

The amylose content of different legume starches varied from 30.61% to 33.55%, and the highest amylose content was found in red adzuki starch, whereas the lowest amylose content was found in faba bean starch. These values were similar with the values previously reported for black gram (30.33%–40.64%; Wani, Sogi, & Gill, 2015), but relatively lower than those reported for chickpeas (46.5%) and pinto beans (52.4%; Yañez‐Farias, Moreno‐Valencia, Falcón‐Villa, & Barrón‐Hoyos, 1997), and field peas (42.9%–43.7%; Ratnayake, Hoover, Shahidi, Perera, & Jane, 2001). The different amylose content among different starch varieties could be attributed to different enzyme activity that involved in the biosynthesis of starch molecules within the starch granules (Krossmann & Lloyd, 2000), and the soil type and climatic conditions during growth and granule size distribution (Singh, Mc‐Carthy, & Singh, 2006). The starch granules with high level of amylose always exhibited a slow hydrolysis rate and a rapid retrogradation rate than starch with low level of amylose.

### 3.2 Morphology characteristic of legume starches

Morphological characteristics of different legume starch granules were observed using scanning electron microscope (SEM), light microscope (LM), polarized light microscope (PLM), and confocal laser scanning microscope (CLSM), and the micrographs are shown in Figure 1. The various types of starch granules exhibited a similar oval, spherical, kidney, and elliptical shape, and the surfaces of all starch samples appeared to be smooth and showed no evidence of fissures (Figure 1a_2_–d_2_). Among all starches, red adzuki bean and baiyue bean starch had the largest angular granules, with the granule diameter ranged from 20 to 40 μm, while the chickpea and faba bean showed a relatively smaller diameter with the granule size ranged between 10 and 30 μm (Figure 1a_2_–d_2_). These observations are mostly in agreement with several other legume starch granule shapes and size (Li, Xiao, Guo, et al., 2014; Ratnayake et al., 2001; Wani et al., 2015). Starch granule shapes and size have been acknowledged to have an influence on the physicochemical and functional properties of starch.

The shapes and size of starches under LM were similar to those under SEM, but many fissures or cracks could be observed on the surface of some granules for these starch varieties (Figure 1a_3_–d_3_). The fissures structure could also be observed on mung bean starch under light microscope (Li, Shu, Zhang, & Shen, 2011). Cracks have been attributed to low granule integrity resulting from suboptimal packing of amylopectin double helices (Blennow et al., 2003). In addition, during processing, it is easy to facilitate rapid water or enzyme penetration of fissured starch surfaces (Maache‐Rezzoug & Allaf, 2005). The various legume starch granules showed a distinct “Maltese crosses” with the hilum in the center of granules under PLM (Figure 1a_4_–d_4_), indicating the high degree of molecular orientation within starch granules. The “maltese cross” under PLM suggests the radial arrangement of amylopectin crystallites within the granule at right angles to the surface with their single reducing end group toward the hilum, and the hilum is the original growing point of the starch granule (Maaran et al., 2014).

Confocal laser scanning microscopy images were used to reveal the distribution of amylose and amylopectin in starch granules and to characterize growth ring, channels, and pores of starch granules. The CLSM optical images of various legume starch granules stained with APTS are shown in Figure 1a_5_–d_5_. APTS specifically reacts with the reducing end of starch molecules leading to a 1:1 stoichiometric ratio of starch molecule labeling. In general, the APTS fluorescence intensity of starch with high amylose content is greater, since amylose has a much smaller molecule than amylopectin and contains a much higher molar ratio of reducing ends per anhydrous glucose residue than the amylopectin molecules, which lead to a higher by‐weight labeling of amylose (Blennow et al., 2003). Herein, no obvious difference in APTS fluorescence intensity could be observed among various legume starches (Figure 1a_5_–d_5_), indicating their similar amylose molecular content as provided in Table 1.

The growth rings, hilum, cracks, and inner channels could be clearly identified among various starch granules. It could be seen that the morphological structure of inner channel and hilum differs significantly for the starches with different original source (Figure 1a_5_–d_4_), and more channels are observed in chickpea starch (Figure 1b_5_). The amylopectin molecules are prominently located in the semi‐crystalline growth rings, while amylose has been accepted to be mainly located within the amorphous zones of the two distinct granule domains (Pérez & Bertoft, 2010). However, the precise role of amylose and amylopectin on the structure of the crystalline cluster structures, as well as the mechanism of the growth ring formation, is still not fully understood. The starch granular pores and channel structure have been always being considered to facilitate the access of enzyme, water, and different solvent to the granule interior.

### 3.3 X‐ray diffraction analysis of legume starches

**Figure 2:**
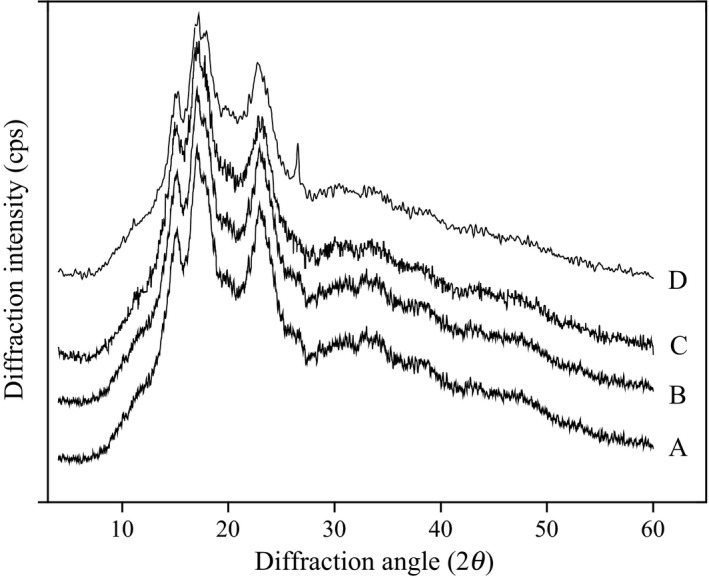
X‐ray diffraction patterns of red adzuki bean (A), baiyue bean (B), faba bean (C), and chickpea (D) starches

The crystal packing arrangements of four kind legume starches were investigated using an X‐ray diffractometer, and the X‐ray diffractograms (XRD) of starch samples are shown in Figure 2. It is convenient to reveal the presence and characteristic of starch crystalline structure by using XRD, and there are usually A‐type, B‐type, and C‐type starches by XRD spectra. There was a strong reflection at 2θ about 15^o^ and 23^o^ and an unresolved doublet at about 17^o^ and 18^o^ in A‐type starch; however, in B‐type starch, the strong diffraction peak present at 17^o^ and a few small peaks at 2θ around 15^o^, 20^o^, 22^o^, and 24^o^, and a characteristic peak at 2θ about 5.6^o^. Nevertheless, C‐type starch is a mixture of both A‐type and B‐type polymorphs with intensity peaks at 2θ about 17^o^ and 23^o^ and a few small peaks located at 5.6^o^ and 15^o^ (Cheetham & Tao, 1998). Starches from four legume varieties presented the similar characteristic diffraction peaks at 2θ around 15^o^, 17^o^, and 23^o^, indicating the presence of C‐type crystallinity in different legume starches (Figure 2). The results are consistent with the report on other legume starches (Li, Xiao, Guo, et al., 2014; Maaran et al., 2014; Ratnayake et al., 2001).

The position of strong diffraction peaks and relatively crystallinity degree of different legume starches are presented in Table 2. The positions of the peaks were found to be similar for the various starches. The chickpea starch exhibited the lowest relative crystallinity degree value among various starch granules, which might be due to the difference in the variety. In addition, there were many other factors such as crystal size, distribution of crystalline regions, and orientation of “crystals” within the semi‐crystalline domains, and the extent of polymer interactions could result in different relative crystallinity in starch (Kaur et al., 2013).

**Table 2:** X‐ray diffraction data of starches isolated from various legume^a^

Varieties	Diffraction peaks at 2θ values (^o^)			Degree of crystallinity (%)
	15^o^	17^o^	23^o^	
Red adzuki bean	15.24	17.20	23.12	18.79 ± 0.49 a
Baiyue bean	14.98	17.16	22.92	17.75 ± 0.77 ab
Faba bean	15.18	17.04	23.18	18.50 ± 0.73 a
Chickpea	15.14	17.20	23.42	16.20 ± 0.52 b

a Means ±* SD* within columns with different letters for same variety are significantly different (*p *<* *0.05).

### 3.4 Swelling power and solubility of legume starches

The swelling power and solubility of the legume starches were investigated over the temperature range of 50–90°C, and the results are presented in Table 3. Regardless of the starch varieties, both the swelling power and solubility of starches sharply increased after the temperature increased in range of 50–90°C. Solubility is generally related to the presence of soluble molecules such as amylose (Tester & Morrison, 1990), which indicates the ability of starch solids to dissolve or disperse in an aqueous solution. Among starch samples, red adzuki starch showed the lowest solubility values at 50°C and the highest solubility values at 90°C compared to other three starch varieties. Faba bean starch showed the lowest solubility values at 90°C (Table 3), and the lower solubility was due to the integrity of the starch granules where restricted swelling and solubility were present. Meanwhile, the high solubility means that the hydrogen bonds can be easily weakened in the starch granules with high amylopectin content. For all legume starches, a rapid increment of solubility was shown after 70°C (Table 3), a pattern that is due to the higher disorganization of starch granules around the gelatinization temperature.

**Table 3:** Swelling power and solubility of starches isolated from various legume^a^

Properties	Varieties	Temperature (^o^C)				
		50	60	70	80	90
Swelling power (g/g)	Red adzuki	0.81 ± 0.18 cd	0.91 ± 0.29 c	3.56 ± 0.23 d	6.87 ± 0.50 c	10.40 ± 0.06 d
	Baiyue	3.38 ± 0.12 a	3.66 ± 0.05 a	4.58 ± 0.1 c	6.84 ± 0.28 c	11.26 ± 0.29 c
	Faba bean	1.09 ± 0.05 b	1.13 ± 0.19 c	6.85 ± 0.15 b	10.29 ± 0.36 b	12.67 ± 0.02 b
	Chickpea	0.78 ± 0.20 d	1.81 ± 0.03 ab	10.60 ± 0.12 a	16.32 ± 0.25 a	18.25 ± 0.06 a
Solubility (%)	Red adzuki	2.14 ± 0.11 c	2.48 ± 0.05 c	5.50 ± 0.10 c	9.50 ± 0.10 ab	14.81 ± 0.20 a
	Baiyue	2.85 ± 0.12 a	3.06 ± 0.11 a	5.47 ± 0.28 c	7.33 ± 0.05 c	10.21 ± 0.14 c
	Faba bean	2.29 ± 0.05 b	2.71 ± 0.07 b	6.67 ± 0.15 b	8.18 ± 0.03 b	9.92 ± 0.09 c
	Chickpea	2.15 ± 0.05 bc	3.38 ± 0.02 a	7.78 ± 0.19 a	10.84 ± 0.07 a	12.62 ± 0.06 b

a Values within the same column for the same wheat genotype followed by a common letter are not significantly different (*p *<* *0.05). The data are presented as mean ± *SD* (triplicate analysis).

Swelling power is a measure of the ability of the starch to hydrate under specific conditions such as temperature and water content, and the greater swelling capacity of the starch granules is, the weaker of the binding forces (Hoover & Manuel, 1996). Baiyue bean starch exhibited higher swelling power than the other three kinds of starches at low temperature (50–60°C). Also, chickpea starch exhibited the highest swelling power at high temperatures (70–90°C) in this study. Again, a dramatic increase in the swelling power in all starch samples could be observed above 70°C. The rapid increase in swelling power at 70–90°C may be due to an increase in molecular mobility of the amorphous region, which causes unraveling, and melting of the double helices present within the amorphous and crystalline domains (Ratnayake et al., 2001). The different swelling power among various starch granules may be related on the amylose–lipid complexes, the intra‐ and intermolecular interactions, and the ratio and molecular weights of amylose and amylopectin (Hoover & Manuel, 1996; Tester & Morrison, 1990), and amylose always acts as an inhibitor of starch swelling.

### 3.5 Light transmittance of legume starches

The light transmittance (turbidity, clarity, or turbidity) of the starch pastes of the starches from the different legume varieties for a period of 3 days is shown in Table. 4. The light transmittance of all gelatinized legume starch paste was found to be decreased with the increase in the storage time period. Red adzuki starch had the highest transmittance during the whole storage period (12–72 hr) among the legume varieties, which may be attributed to its lowest amylose content (Table 1). Considerable reduction in light transmittance was observed up to the 48 hr of storage for all samples and then persisted in a slight reduction rate after that. The decrease in clarity was a result of retrogradation of starch paste. During the storage period, the gelatinized starch molecules including amylose and amylopectin reassociate into an ordered structure to retrieve a crystalline order, which resulted in decrease in transmittance.

**Table 4:** Light transmittance of starches isolated from various legume^a^

Varieties	Light transmittance (%)						
	0 hr	12 hr	24 hr	36 hr	48 hr	60 hr	72 hr
Red adzuki	7.43 ± 0.05 b	4.76 ± 0.28 a	4.05 ± 0.28 a	2.27 ± 0.06 a	1.63 ± 0.03 a	1.55 ± 0.14 a	1.22 ± 0.06 a
Baiyue	2.32 ± 0.00 d	2.16 ± 0.00 d	1.50 ± 0.01 c	1.34 ± 0.0 c	0.98 ± 0.00 c	0.63 ± 0.09 d	0.47 ± 0.04 d
Faba bean	3.84 ± 0.02 c	2.74 ± 0.03 c	2.25 ± 0.04 b	1.65 ± 0.04 b	1.18 ± 0.01 b	1.05 ± 0.02 b	0.83 ± 0.02 b
Chickpea	8.97 ± 0.15 a	3.46 ± 0.37 b	2.25 ± 0.06 b	1.20 ± 0.07 d	0.87 ± 0.02 d	0.77 ± 0.01 c	0.68 ± 0.01 c

a Values within the same column for the same wheat genotype followed by a common letter are not significantly different (*p *<* *0.05). The data are presented as mean ± *SD* (triplicate analysis).

The rapid decrease in clarity during the early storage may be due to the aggregation and crystallization of amylose, and then, the decrease rate in clarity during the rest of the storage could be attributed to the leached amylose and amylopectin chains that lead to the development of the functional zone which scatters a significant amount of light (Yu, Ma, Menager, & Sun, 2012). It has been reported that the difference in light transmittance among various starch granules is related to different granule remnants, granule swelling, leached amylose and amylopectin, amylose and amylopectin chain lengths, intra‐ or intermolecular bonding, lipids, and substitution (Jacobson, Obanni, & BeMiller, 1997). As for legume starches, the legume starches contained varying amounts of phosphate monoester substituents might also contribute to variation in starch gel clarity (Jane, Kasemsuwan, & Chen, 1996).

### 3.6 Thermal properties of legume starches

**Figure 3:**
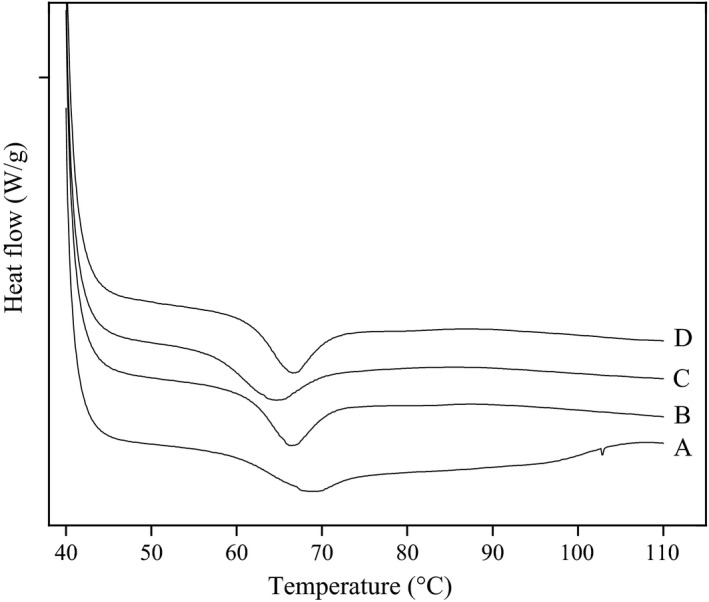
Differential scanning calorimeter endotherms of starches extracted from red adzuki bean (A), baiyue bean (B), faba bean (C), and chickpea (D)

The gelatinization thermograms of starches isolated from different legume varieties are shown in Figure 3, and the thermal transition temperatures onset transition temperature (*T*_o_), peak temperature (*T*_p_), conclusion transition temperature (*T*_c_), gelatinization temperature range (Δ*T*_r_) and gelatinization enthalpy (Δ*H*) on a dry starch basis are presented in Table 5. The DSC endothermic peak is linked to starch gelatinization which can reflect the loss of ordered structures (double helices/crystallites) of starch granules (Cooke & Gidley, 1992). The gelatinization transition parameters and gelatinization enthalpy of starches from various legume varieties differed significantly as shown in Table 5. Gelatinization temperature is a reflection of the perfectness of starch crystallites (Tester & Morrison, 1990). The *T*_o_ of legume starches ranged from 58.73 to 61.96°C, *T*_p_ in the range of 64.07–68.35°C, and *T*_c_ in the range of 71.30–78.99°C, and the chickpea starch showed the lowest *T*_o_, *T*_p,_ and *T*_c_ values among various legume starches (Table 5). The lower gelatinization transition temperature of chickpea starch is indicative that its crystallites have a lower degree of stability (reflects looser packing of amylopectin crystallites within the crystalline lamella) and/or more structural defects. In addition, it has been reported that the lower transition temperature and gelatinization enthalpy have a relationship with the smaller amount of long‐chain amylopectin molecules and greater amount of short‐chain amylopectin (Inouchi, Ando, Asaoka, Okuno, & Fuwa, 2000; Jane et al., 1999).

**Table 5:** Gelatinization parameters of different legume starches as determined by DSC^a^^,^ b

Varieties	*T*_o_ (^o^C)	*T*_p_ (^o^C)	*T*_c_ (^o^C)	Δ*T*_r_ (^o^C)	Δ*H* (J/g)
Red adzuki bean	61.22 ± 0.78 a	68.35 ± 0.22 a	78.99 ± 0.81 a	17.66 ± 0.69 a	6.76 ± 0.05 a
Baiyue bean	61.61 ± 0.44 a	66.24 ± 0.17 b	73.00 ± 0.08 b	11.44 ± 0.17 c	7.06 ± 0.13 a
Faba bean	61.96 ± 0.09 a	66.38 ± 0.19 b	73.06 ± 0.00 b	11.09 ± 0.21 c	6.68 ± 0.07 a
Chickpea	58.73 ± 0.08 b	64.07 ± 0.11 c	71.30 ± 0.35 c	12.50 ± 0.22 b	5.57 ± 0.06 b

^a^All values are means of triplicate determinations ± *SD*. Means within columns with different letters are significantly different (*p *<* *0.05). ^b^ *T*_o_, onset temperature; *T*_p_, peak temperature; *T*_c_, conclusion temperature; Δ*T*_r_, gelatinization temperature range (Δ*T*_r _= *T*_c_−*T*_o_); Δ*H*, enthalpy of gelatinization.

The DSC curves of red adzuki bean starches exhibited wider peaks than those from other three legume starch (Figure 3), which suggested its broader range of gelatinization temperature (Δ*T*_r_) as shown in Table 5. The Δ*T*_r_ is related to the heterogeneity of the starch granule sizes and the distribution of amylose and amylopectin, which produce a semi‐crystalline arrangement inside the granule (Agama‐Acevedo et al., 2014). A wide melting range might imply crystals with a large variation in stability, whereas a narrow range could suggest crystals of a more homogeneous quality and similar stability. The lower Δ*T*_r_ value of red adzuki bean starch may be attributed to the higher homogeneity of crystallites within starch granules. The Δ*H* was observed to be highest for baiyue bean starch, whereas it was lowest for chickpea starch. The Δ*H* gives an overall measure of crystallinity and is an indicator of the loss of molecular order within the granule during gelatinization (Cooke & Gidley, 1992). The lower Δ*H* indicates a lower degree of organization in or a lower stability of the crystals (Chiotelli & Meste, 2002). Red adzuki bean and faba bean starch had broader gelatinization temperature ranges and higher gelatinization enthalpy than other starch samples (Table 3). These results are in agreement with the observation that red adzuki bean and faba bean starch had a higher relative crystallinity degree (Table 2) and required more energy for gelatinization.

### 3.7 Pasting properties

**Figure 4:**
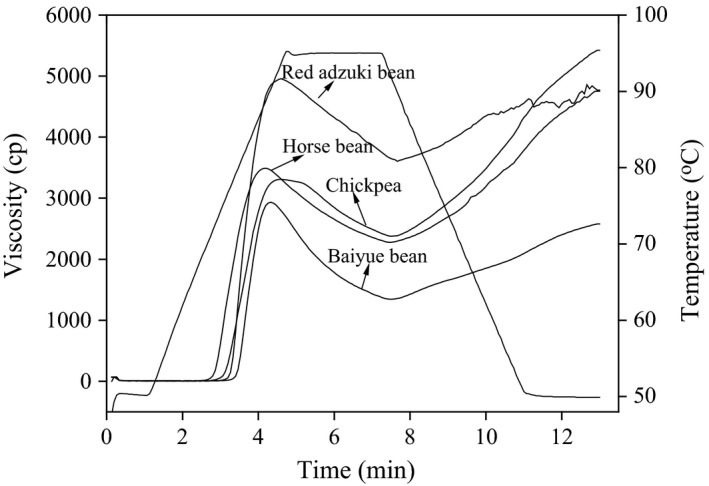
Pasting profile of starches extracted from different legume varieties

Pasting viscosity profiles of starches isolated from various legumes are illustrated in Figure 4, and the related parameters are summarized in Table 6. The overall shape of the RVA pasting curves was similar for the four kinds of legume starches (Figure 4). However, there were significant differences (*p* < 0.05) in PV, trough viscosity (TV), breakdown (BD), final viscosity (FV), setback (SB), peaking time (PT), and pasting temperature (GT) between the starches (Table 6). Changes in viscosity during heating process give an indication of the starch stability, and the changes occurring during cooling show the consistency of the product when consumed (Kaur, Sandhu, & Lim, 2010). Peak viscosity of rice starch samples ranged from 2657 to 3911 cP, and the highest PV was shown by red adzuki bean starch, whereas the lowest PV was showed by baiyue bean starch. Peak viscosity refers to the maximum viscosity attained by gelatinized starch during heating in water; in the meantime, it can also indicate the water‐binding capacity of the starch granule (Shimelis, Meaza, & Rakshit, 2006). The difference in PV among various legume starches could be due to variation in the water absorption rate and starch swelling properties during heating.

**Table 6:** Pasting properties of starches isolated from different legume varieties^a^

Varieties	PV (cP)	TV (cP)	BD (cP)	FV (cP)	SB (cP)	PT (min)	GT (^o^C)
Red adzuki	4,930 ± 34 a	3,605 ± 21 a	1,325 ± 22 b	4,764 ± 101 b	1,159 ± 123 c	4.6 ± 0.0 a	75.6 ± 0.5 b
Baiyue	2,949 ± 26 d	1,360 ± 18 d	1,589 ± 7 a	2,573 ± 8 d	1,213 ± 26 c	4.37 ± 0.1 c	77.5 ± 0.1 a
Faba bean	3,524 ± 60 b	2,277 ± 12 c	1,247 ± 49 b	4,814 ± 58 b	2,536 ± 55 b	4.2 ± 0.0 d	70.2 ± 0.0 d
Chickpea	3,286 ± 29 c	2,367 ± 14 b	919.5 ± 14 c	5,378 ± 65 a	3,011 ± 52 a	4.53 ± 0.0 b	73.1 ± 0.5 c

Notes

BD: breakdown; FV: final viscosity; GT: pasting temperature; PT: peak time; PV: peak viscosity; SB: setback; TV: trough viscosity.

a All values are means of triplicate determinations ± *SD*. Means within columns with different letters are significantly different (*p *<* *0.05).

Trough viscosity was the highest for red adzuki bean starch and the lowest for baiyue bean starch, and BD viscosity was the highest for baiyue bean starch and the lowest for chickpea starch. TV has been reported to be influenced by the extent of amylose leaching, amylose–lipid complex formation, friction between swollen granules, granule swelling, and competition between leached amylose and remaining granules for free water (Olkku & Rha, 1978). Breakdown is an indication of the degree of its organization within the starch granule and a tendency to lose viscosity upon holding and shearing. It has been reported that the higher the breakdown viscosity value is, the lower the ability of the starch granules to withstand heating and shear stress during cooking (Adebowale, Olu‐Owolabi, Olawunmi, & Lawal, 2005). Thus, the low breakdown value of chickpea starch indicates its restricted starch granule swelling and slow tendency to lose viscosity, which might be able to withstand more heating and shear stress than other starch varieties that possess higher breakdown values.

The FV value varied from 2573 cP to 4764 cP, and the highest FV was recorded for chickpea starch and the lowest for baiyue bean starch. FV can show the ability of starch to form a viscous paste, which is largely influenced by the retrogradation tendency of the soluble amylose during cooling stage (Olkku & Rha, 1978). The higher FV of the faba bean and chickpea starch indicated that they have a higher tendency to retrograde after cooling due to the recrystallization of leached amylose molecules. The setback viscosity gives an indication of the recrystallization of gelatinized starch during cooling. Setback viscosity of the legume starches varied from 1159 cP (red adzuki starch) to 3011 cP (chickpea starch), and this indicated that chickpea starch might have a greater retrogradation tendency than other starch varieties. Pasting temperature is the temperature at which the viscosities of the starch pastes begin to rise. The GT of various starch varieties ranged from 70.2 to 77.5°C, and red adzuki starch and baiyue starch showed higher GT than the other two varieties. The high GT of red adzuki starch and baiyue starch indicates that these two starches have a higher resistance to swelling and rupture. Also, the peak time for red adzuki starch was significantly higher than other varieties. Additionally, the higher GT and peak time of red adzuki starch suggested higher energy costs would be required during cooking. The RVA analysis confirmed that starches from different legumes have distinct cooking behaviors and hence different possible applications.

## 4 CONCLUSION

Structural and physicochemical properties of starches from red adzuki bean, chickpea, faba bean, and baiyue bean starch were characterized, and a high variability among various legume starches properties was found. There were minor differences in morphology characteristic properties among various legume starches. The X‐ray diffraction patterns of all legume starches showed C‐type pattern with relative crystallinity degree varied among the varieties. The swelling power, solubility, light transmittance, and gelatinization parameter values of legume starches varied among the varieties. Pasting properties significantly varied and are useful for assessing the cooking quality of legume starches. Differences in physicochemical properties were influenced by the differences in morphology, composition, and structure of various legume starches. The findings would be useful in selecting legume variety or legume starch for specific food and ingredient applications, and also for further investigations on fine structure of starch granules and modifications targeting food ingredient applications.
