# Correction to “Compositional, Morphological, and Physiochemical Properties of Starches From Red Adzuki Bean, Chickpea, Faba Bean, and Baiyue Bean Grown in China”

**DOI:** 10.1002/fsn3.4709

**Published:** 2024-12-19

Zhang, Z. S., Tian, X. L., Wang, P., Jiang, H., & Li, W. H. (2019). Compositional, morphological, and physicochemical properties of starches from red adzuki bean, chickpea, faba bean, and baiyue bean grown in China. *Food Science & Nutrition*, 7(8), 2485–2494. https://doi.org/10.1002/fsn3.865.

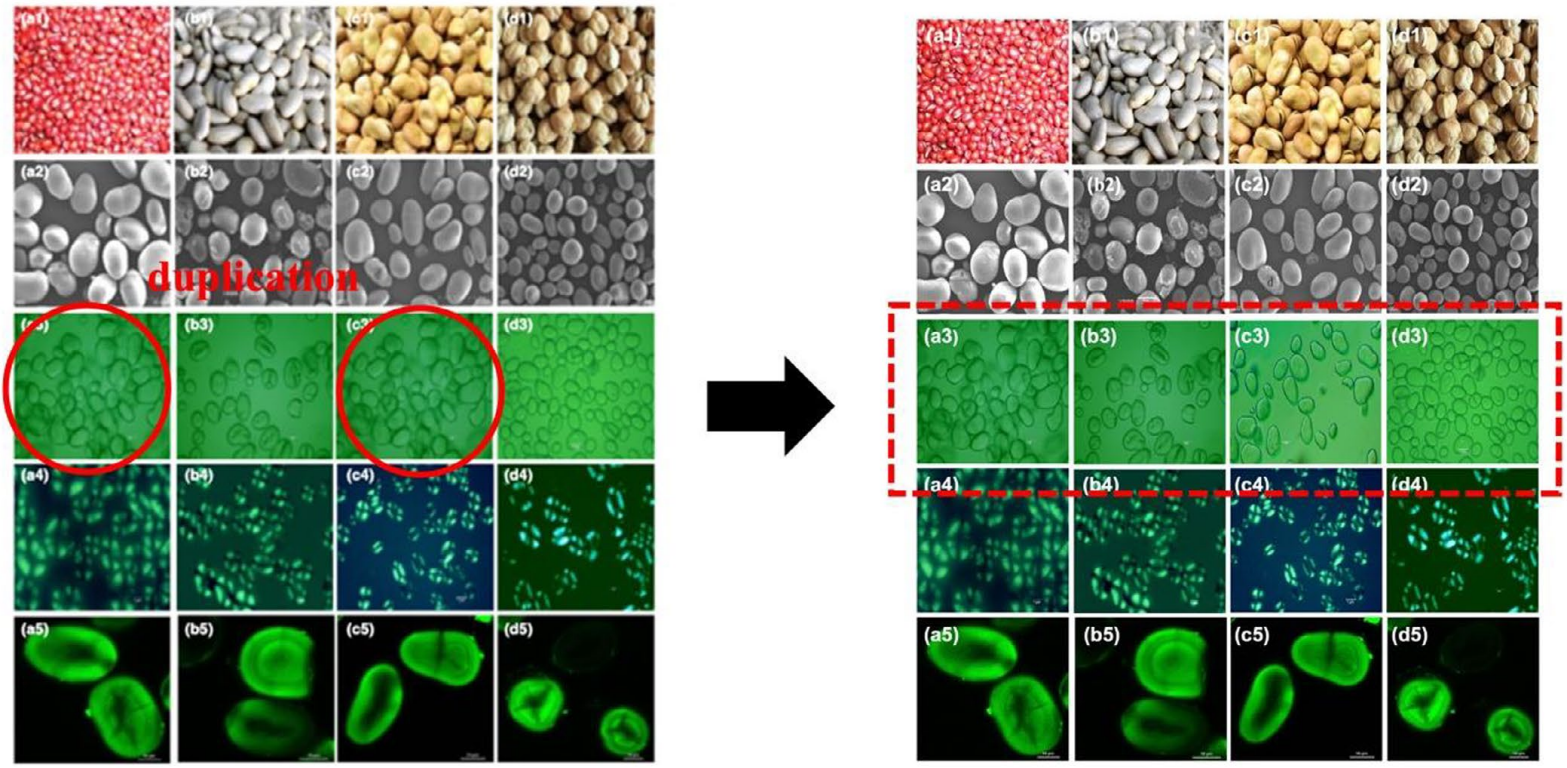

In Figure 1, the a3 and c3 images in the light microscope micrographs were duplicates. The corrected figure appears below. This error does not affect any of the other elements or the presentation and interpretation in the Results and Discussion.

We apologize for this error.

